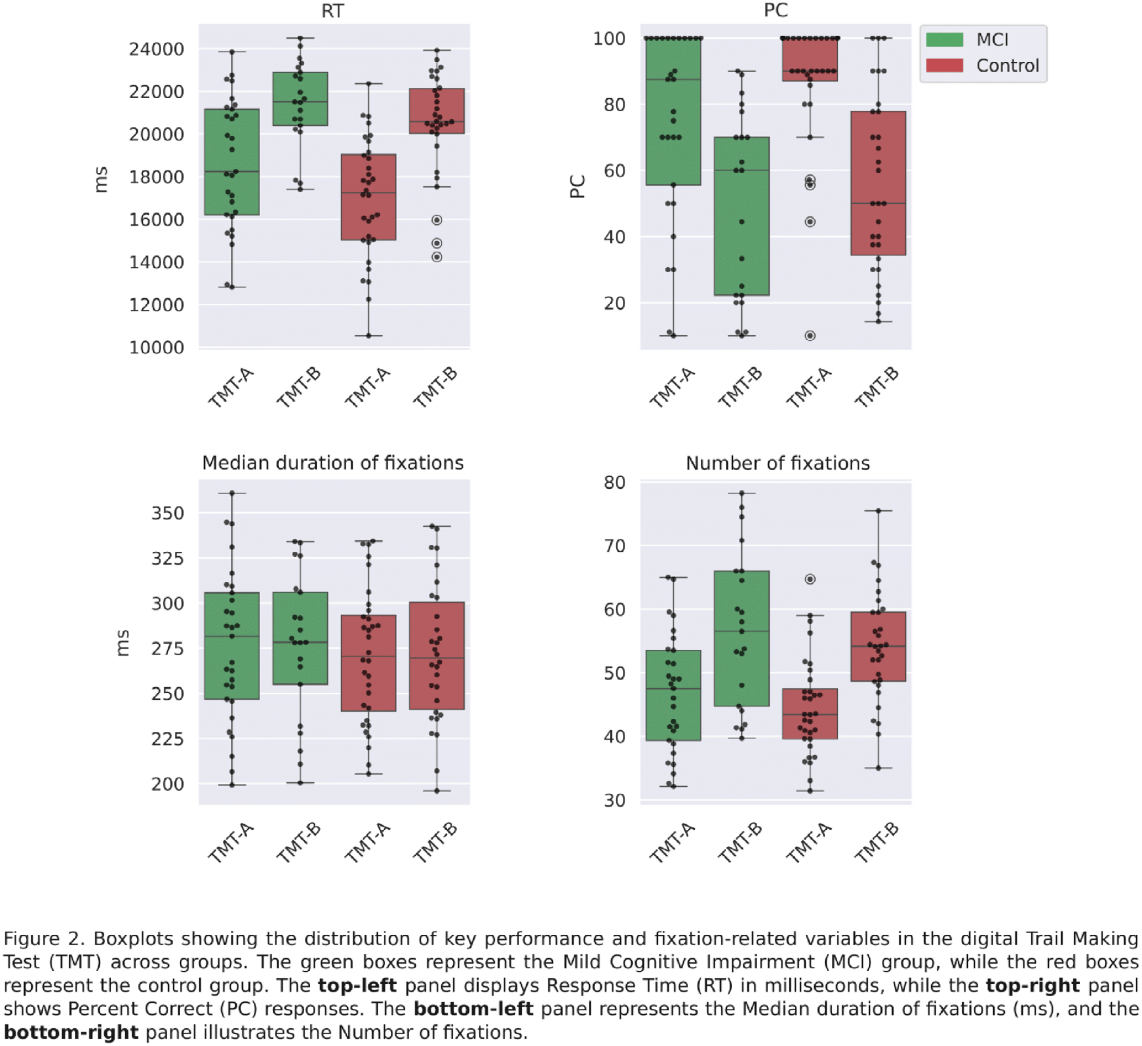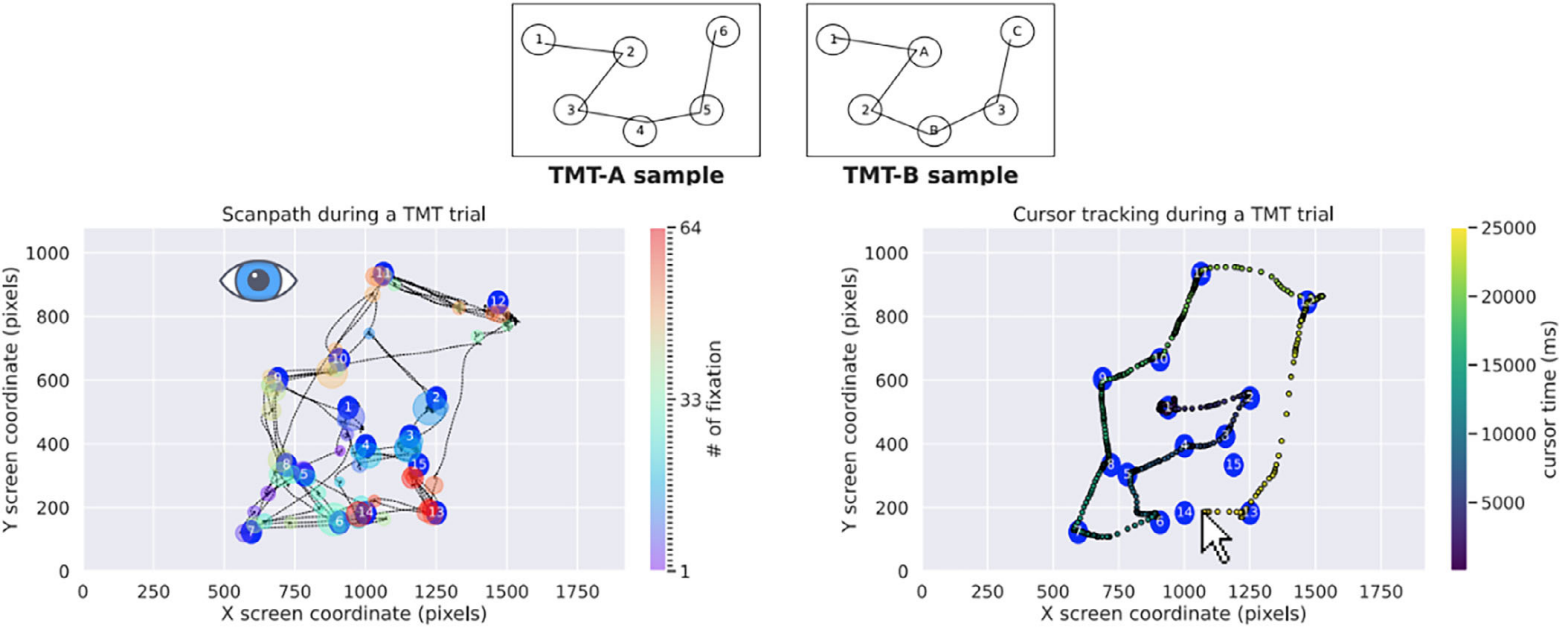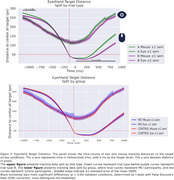# Enhancing MCI Assessment: A Digital Trail Making Test with Integrated Eye and Hand Tracking

**DOI:** 10.1002/alz70856_107204

**Published:** 2026-01-10

**Authors:** Gustavo E Juantorena, Waleska Berrios, Maria Cecilia Fernández, Agustin Ibanez, Agustin Petroni, Juan E Kamienkowski

**Affiliations:** ^1^ Instituo de Ciencias de la Computacion (CONICET‐UBA), Ciudad Autonoma de Buenos Aires, Ciudad Autonoma de Buenos Aires, Argentina; ^2^ Facultad de Ciencias Exactas y Naturales, Universidad de Buenos Aires, Ciudad Autonoma de Buenos Aires, CABA, Argentina; ^3^ Churruca Visca Hospital, Buenos Aires, Argentina; ^4^ Complejo Médico hospitalario Churruca Visca, buenos aires, Argentina; ^5^ Hospital Italiano de Buenos Aires, Buenos aires, Argentina; ^6^ Global Brain Health Institute, University of California, San Francisco, USA; ^7^ Center for Social and Cognitive Neuroscience (CSCN), School of Psychology, Universidad Adolfo Ibañez, Santiago, Chile; ^8^ Latin American Brain Health Institute (BrainLat), Universidad Adolfo Ibañez, Santiago, Chile; ^9^ Universidad Autónoma de Caribe, Barranquilla, Colombia; ^10^ Global Brain Health Institute (GBHI), University of California San Francisco (UCSF); & Trinity College Dublin, Dublin, Leinster, Ireland; ^11^ Trinity College, Dublin, Ireland; ^12^ National Scientific and Technical Research Council (CONICET), Buenos Aires, Argentina; ^13^ BrainLat Institute, Santiago, Santiago, Chile; ^14^ Cognitive Neuroscience Center (CNC), Universidad de San Andrés, Buenos Aires, Buenos Aires, Argentina; ^15^ Latin American Brain Health Institute (BrainLat), Universidad Adolfo Ibáñez, Santiago, Región Metropolitana de Santiago, Chile; ^16^ Trinity College Dublin, Dublin, Leinster, Ireland; ^17^ Universidad Adolfo Ibanez, Santiago de Chile, Chile; ^18^ Universidad de San Andrés, Buenos Aires, Argentina; ^19^ Instituto de Neurociencia Cognitiva y Traslacional (INCyT), Buenos Aires, Argentina; ^20^ Latin American Institute for Brain Health (BrainLat), Universidad Adolfo Ibañez, Santiago, Chile; ^21^ University of Gothenburg, Gothenburg, Gothenburg, Sweden; ^22^ Facultad de Ciencias Exactas y Naturales, Universidad de Buenos Aires, Ciudad Autonoma de Buenos Aires, Ciudad Autonoma de Buenos Aires, Argentina

## Abstract

**Background:**

We extended our computerized Trail Making Test (c‐TMT) to investigate deficits in Mild Cognitive Impairment (MCI) compared to neurotypical controls. By integrating hand and eye tracking, we captured fine‐grained movement dynamics, revealing distinct trajectory alterations in MCI patients. These differences suggest potential digital biomarkers, offering a more precise assessment beyond traditional total time measurements.

**Methods:**

Twenty‐nine MCI patients and 28 age‐ and education‐matched controls (with significant Mini‐Mental Test differences, *p* < 0.001) were enrolled at Hospital Italiano de Buenos Aires, Argentina, with informed consent. Two practice trials and 20 experimental trials (alternating TMT‐A and TMT‐B) were presented. Stimuli were displayed on a 24‐inch screen. Gaze was recorded from the right eye at 500 Hz using an EyeLink 1000 Plus. The mouse trajectory was displayed in real‐time, with feedback on the correct element selection.

**Results:**

Linear Mixed Models (LMM) were applied to correct trials to estimate the main effects of subject group (MCI vs. control), trial type (TMT‐A vs. TMT‐B), and their interaction using the statsmodels library in Python. For performance metrics, LMM revealed a significant effect of subject group and trial type on the percentage of completion (PC) (SE = 0.066, *p* = 0.040; SE = ‐9.017, *p* = 1.9 × 10^−19^) and the time required to complete a trial (RT) (SE = ‐2.514, *p* = 0.012; SE = 7.896, *p* = 2.9 × 10^−15^). For eye‐tracking metrics, we found significant differences for both trial type (SE = 2.06, *p* = 0.002) and subject group (SE = 2.81, *p* = 0.023) in scanpath length (number of fixations). However, fixation duration differences were not significant (SE = 7.830, *p* = 0.68; SE = 12.90, *p* = 0.80). We also analyzed eye‐hand coordination by parsing fixations based on mouse position and time‐locking mouse and hand movements to target entry. Differences were observed by trial type but not by subject group.

**Conclusions:**

Our c‐TMT version identified significant differences in scanpath length between MCI patients and controls. Hand and eye movements together allow fixation analysis to determine how increased fixations are distributed. These findings highlight the potential of this approach in Digital Neuropsychology.